# Our Colleges

**Published:** 1896-08

**Authors:** 


### OUR COLLEGES.

THE opening sessions of our veterinary schools for 1896 will mark an important epoch in the history of veterinary education in America, and those who for the first time step forward on a broader basis and more advanced curriculum deserve well the support of every veterinarian throughout the land. The earnest commendation and encouragement of the United States Veterinary Association should be theirs, and the strong arm of all local organizations should be placed about them in support of their efforts. The leaders in this movement have never looked backward after the first step, and their faith and courage have now brought new recruits to the ranks, and they should add strength and position to all. These changes, be it remembered, have taken place at a time when, from a financial point of view, they will be severely felt, for retrenchment in every line of industry throughout our land has prevailed for over two years, and the schools and colleges of every profession and vocation have felt keenly the results thereof. This then is the hour for the profession to lend in every way their aid and assistance, their good-will and support, their recognition and kind word of encouragement, that the added strength may in part compensate for the sacrifices made, and that we may hope to win over the few remaining institutions that should be within the fold.

---

As we close the last forms prior to going to press we are favored with the glad news that the National Veterinary College will hereafter be the Veterinary Department of the Columbian University. It will enjoy all the laboratory facilities and the aid of such of the corps of instructors as may be needed. This is a grand step and will aid this school in becoming one of the foremost in the country. Entering for the first time on her obligatory course of three years, she will now be afforded means and power to better demonstrate the worth of the teachers already associated with this school, and thus give to the profession well-equipped men as her graduates.

Surely the signs of the times are auspicious.

On to Buffalo!

